# Effect of Initial Microstructure on the Toughness of Coarse-Grained Heat-Affected Zone in a Microalloyed Steel

**DOI:** 10.3390/ma14164760

**Published:** 2021-08-23

**Authors:** Minghao Shi, Man Di, Jian Zhang, Rangasayee Kannan, Jing Li, Xiaoguang Yuan, Leijun Li

**Affiliations:** 1 School of Material Science and Engineering, Shenyang University of Technology, Shenyang 110870, China; Dm1660252588@126.com (M.D.); yuanxg@sut.edu.cn (X.Y.); 2 Department of Chemical and Materials Engineering, University of Alberta, Edmonton, AB T6G 1H9, Canada; rangasay@ualberta.ca (R.K.); jing20@ualberta.ca (J.L.); leijun@ualberta.ca (L.L.); 3 Ansteel Beijing Research Institute, Beijing 102211, China; mjjt_2014@126.com; 4 Materials Science and Technology Division, Oak Ridge National Laboratory, Oak Ridge, TN 37831-6064, USA

**Keywords:** initial microstructure, coarse-grained heat-affected zone, prior austenite grain size, Charpy impact toughness, grain boundary ferrite

## Abstract

Toughness of the coarse-grained-heat-affected-zone (CGHAZ) strongly depends on the prior austenite grain size. The prior austenite grain size is affected not only by chemical composition, thermal cycle, and dissolution of second-phase particles, but also by the initial microstructure. The effect of base metal microstructure (ferrite/pearlite obtained by air cooling and martensite obtained by water-quenching) on Charpy impact toughness of the CGHAZ has been investigated for different heat inputs for high-heat input welding of a microalloyed steel. A welding thermal cycle with a heat input of 100 kJ/cm and 400 kJ/cm were simulated on the MMS-300 system. Despite a similar microstructure in the CGHAZ of both the base metals, the average Charpy impact energy for the air-cooled base metal was found to be higher than the water-quenched base metal. Through thermo-kinetic simulations, it was found that a higher enrichment of Mn/C at the ferrite/austenite transformation interface of the CGHAZ of water-quenched base metal resulted in stabilizing austenite at a lower A1 temperature, which resulted in a coarser austenite grain size and eventually lowering the toughness of the CGHAZ.

## 1. Introduction

The balance between strength and toughness in high-strength low-alloy steels can be disrupted by welding, which is characterized by rapid heating and cooling, producing the heat-affected zone (HAZ), especially the coarse-grained heat-affected zone (CGHAZ) adjacent to the weld fusion line [1,2,3]. The combination of an austenite grain coarsening and rapid cooling promotes the formation of brittle microstructural constituents, containing high proportions of upper bainite (UB), grain boundary ferrite (GBF) and Widmanstatten ferrite (WF), and martensite-austenite (M-A) modules, which may be detrimental to toughness of the CGHAZ. It is generally believed that a microstructure consisting of acicular ferrite and lower inclusion content provides the optimum strength and toughness for both weld metal and CGHAZ [4,5,6,7].

Recent investigations have been carried out to improve the toughness of the CGHAZ, focusing on enhancing acicular ferrite (AF) with interlocking characteristics, refining the prior austenite grains, and minimizing the formation of M-A constituent. The formation of acicular ferrite is related to the chemical composition of the steel, the prior austenite grain size, heat input, and the cooling rate from 800–500 °C (measured by Δt_8/5_). Oxides act as ferrite nucleation sites on-cooling, promote acicular ferrite, and prevent coarse bainite or martensite [8,9,10]. Oxide forming elements, such as Mg, Ti, and Zr, can be added to the microalloyed steels to enhance acicular ferrite during the austenite-ferrite transformation. Li et al. [11] reported the effect of Mg addition on the formation of acicular ferrite in an Al-killed low carbon steel, showing Mg-containing inclusions inducing the nucleation of AF with interlocking characteristics. Chai et al. [12] studied the nucleation behavior of acicular ferrite in a Ti-killed C-Mn steel using the Gleeble thermo-mechanical simulator. The nucleation of acicular ferrite on inclusions was attributed to the formation of Ti_2_O_3_ as nucleation sites. Liu et al. [13] investigated the effect of Zr additions on microstructure and impact toughness in the CGHAZ of high-strength low-alloy steels simulated with a heat input of 100 kJ/cm at peak temperature of 1350 °C. The results indicated that most acicular ferrite has nucleated on Zr-Al-Ti complex oxides in the CGHAZ. Zhao et al. [14] have studied the effect of austenite grain size on acicular ferrite transformation in a high-strength low-alloy steel. It was found that the reduction of prior austenite grain sizes without deformation from 62.8 μm to 37.0 μm promoted the acicular ferrite transformation. Shi et al. [15] investigated the effect of high heat input on toughness in CGHAZ with a heat input of 100 kJ/cm to 1000 kJ/cm in Zr-bearing low carbon steels. Excellent impact toughness in the CGHAZ simulated with a heat input of 1000 kJ/cm was obtained.

The main factors affecting prior austenite grain sizes are the volume fraction of high melting point particles, such as carbides, nitrides, and oxides [16,17], and the welding thermal cycle, i.e., the peak temperature [18]. Among precipitates, it was found that TiN precipitates with a Ti/N ratio close to two was found to provide more stable prior austenite grains [19]. It was also found that Ti containing precipitates are stable up to a higher peak temperature resulting in controlling grain size up to higher peak temperatures [20]. Zr is also used to control prior austenite grain size with ZrN particles acting as particles to pin the prior austenite grains [21]. Shi et al. [16] reported on the effect of microalloying addition of Zr on the characteristics of inclusions and prior austenite grain sizes following a quenching heat treatment. The average prior austenite grain size of the Zr-containing steel is consistently smaller than that of the Zr-free steel, due to a large number of fine Zr-oxide inclusions and Ti(C, N) precipitates, pinning the austenite grain boundaries at temperatures up to 1400 °C.

The objective of the present study is to investigate the effect of initial base metal microstructure on prior austenite grain size, and to correlate the heat input, prior austenite grain size, room temperature microstructure with impact toughness of the CGHAZ. We found the CGHAZ toughness is also affected by the initial microstructure of a Zr-bearing microalloyed steel. We started from two types of prior microstructure, air-cooled and water quenched, of a single base steel, subjected them to the same welding thermal cycle. Significant differences in impact toughness of CGHAZ have been observed, indicating a worse toughness for the water-quenched martensitic initial microstructure. This study provides a new perspective on the microstructure and mechanical properties evolution in the CGHAZ, which is of great significance for submerged-arc welding and additive manufacturing of microalloyed steels.

## 2. Experimental Procedure

The experimental steel was fabricated in a vacuum induction melting furnace. High-purity electrolytic iron was prepared before steelmaking. During steelmaking, the steel melt was shielded in argon. The powder sample was prepared to determine the carbon content by chemical titration. The sample with a diameter of 4 mm and length of 10 mm cylinder was prepared to measure oxygen and nitrogen contents by ON-3000 oxygen and nitrogen analyzer. For metallic elements, the sample was prepared from the experimental steel and machined into a cylindrical shape, with 35 mm diameter and 10 mm length. The analysis of alloy elements was carried out on the Zetium X-ray fluorescence spectrometer (XRF). The chemical composition of the steel is listed in Table 1. The 80 mm × 100 mm × 400 mm ingots were reheated to 1200 °C for 2 h and rolled into 13 mm thick plates by thermo-mechanical control processing in both the recrystallizing and the non-recrystallizing zones. Initially, the ingots were rolled into 45 mm thickness at 1150 °C for 2 passes. After air cooling to 870 °C, the plates were rolled into 13 mm thickness for 5 passes. In order to obtain a different initial microstructure, the plates were cooled by natural cooling and quenched with water to room temperature, respectively, after the last rolling pass.

**Figure 1:**
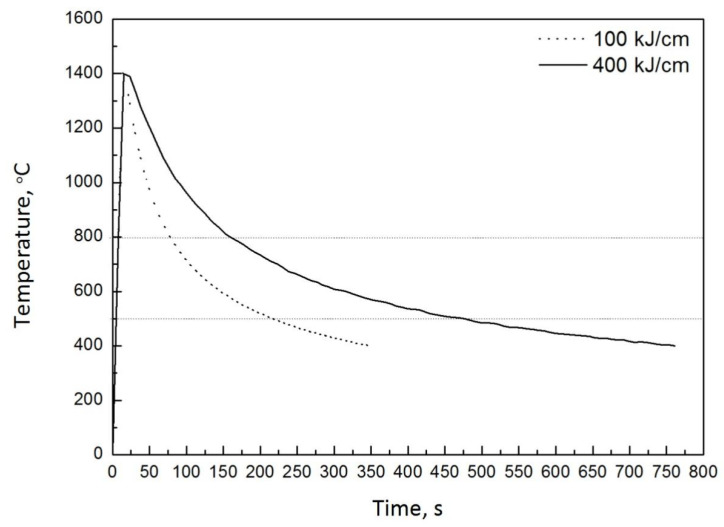
Recorded welding thermal cycles for simulation of the coarse-grained heat-affected zone.

High heat-input welding thermal cycles typical for submerged-arc welding were reproduced on a thermo-mechanical simulator to produce the coarse-grained heat-affected zone (CGHAZ) microstructure. Specimens with two different initial microstructures were cut along the transverse of hot rolling direction of the steel plates and machined into dimensions of 11 mm × 11 mm × 55 mm for CGHAZ simulations. The thermal cycle was characterized by the peak temperature (T_p_), cooling time from 800–500 °C (Δt_8-5_), and the heat input (E). The specimens were rapidly heated to peak temperature (T_p_) of 1400 °C with heating rate of 100 °C/s, holding time of 1 s and 3 s, and the cooling time from 800–500 °C corresponded to 137.5 s and 325 s, which is equivalent to a welding heat input of 100 kJ/cm and 400 kJ/cm used in submerged-arc welding of microalloyed steels, respectively. The recorded thermal cycles are shown in Figure 1.

Specimens cut from hot-rolled steel plates were machined into a cylindrical shape, with 3 mm diameter and 10 mm length. Dilation curves were measured using a FORMASTOR-FII high-speed quench dilatometer (FUJITECH, Japan). FUJITECH, none, good, Japan) from these cylindrical specimens, which were heated to 1300 °C for 1 s with the heating rate of 1 °C/s in N_2_ gas, and then water quenched to room temperature.

Standard round tensile samples with the gauge length of 50 mm and diameter of 8 mm were prepared from hot rolled steel plates along the transverse direction. According to ASTM E-8, the tensile test was performed using a CMT-5105 testing machine (MTS, USA) with a 50 mm gauge length extensometer at room temperature in a cross-head speed of 3 mm/min. Every tensile test was repeated once. According to ASTM E-23, standard Charpy V-notch (CVN) impact samples with the size of 10 mm × 10 mm × 55 mm were prepared. The CVN impact energy was measured at −20 °C by use of an Instron Dynatup 9200 series instrumented drop weight impact tester (Instron, USA). The micro hardness was conducted with a test force of 0.5 kgf and dwell time of 10 s on a Wilson VH3100 hardness tester (Buehler, Germany). Every hardness test was repeated five times in different locations.

For microstructure characterization, the samples were polished by using a Buehler Ecomet™ 250/300 grinder-polisher (Buehler, Germany) with a power head. Grit 320, 600, 1200 SiC sandpapers were used for grinding, 3 μm and 1 μm diamond suspensions were used for polishing, and then etched with 2% nital and 4% picral solutions to reveal the microstructure.

The quenched cylindrical specimens were then characterized for prior austenite grain size. The region near the monitoring thermocouple was prepared using the standard metallographic procedure to reduce the error resulting from variations in peak temperatures and non-uniform cooling rates. In order to reveal prior austenite grain boundaries, a saturated picric acid solution was used to etch the specimens. Prior austenite grain size was measured by the line-intercept method based on low-magnification optical microscopy. All metallographic samples were examined using a light optical microscope and Zeiss Sigma™ FESEM (Zeiss, Germany). All thermodynamic calculations presented were performed using the TCFE9 and MOBFE4 databases on ThermoCalc, DICTRA, and PRISMA. To differentiate martensite and ferrite in ThermoCalc, DICTRA, and PRISMA, for the water quenched base metal, an initial martensitic lath size 2μm (measured from optical images), with enhanced mobility (mobility enhanced by a factor of 10) was used. For the air-cooled base metal, a polygonal ferrite grain size of 10 to 15μm (measured from optical images) without any mobility enhancement was used. Enhanced mobility was used to simulate the martensitic microstructure which inherently has a higher dislocation density, unlike ferrite which has lower dislocation density. ThermoCalc/DICTRA/PRISMA does not take into account the difference between martensite and ferrite, therefore, the mobility was increased by 10 to simulate martensitic microstructure. Considering that the martensite forms by a diffusionless mechanism, martensite composition was set as ferrite with bulk composition of the alloy. For ferrite, equilibrium concentration was estimated from ThermoCalc and used as input for kinetic simulations. DICTRA and PRISMA calculations were performed under zero flux boundary condition with BCC, FCC, and Cementite allowed to form under local equilibrium conditions. To reduce the computational requirement, only four elements, Fe, C, Mn, Si, were considered for the kinetic simulations.

## 3. Results

**Figure 2:**
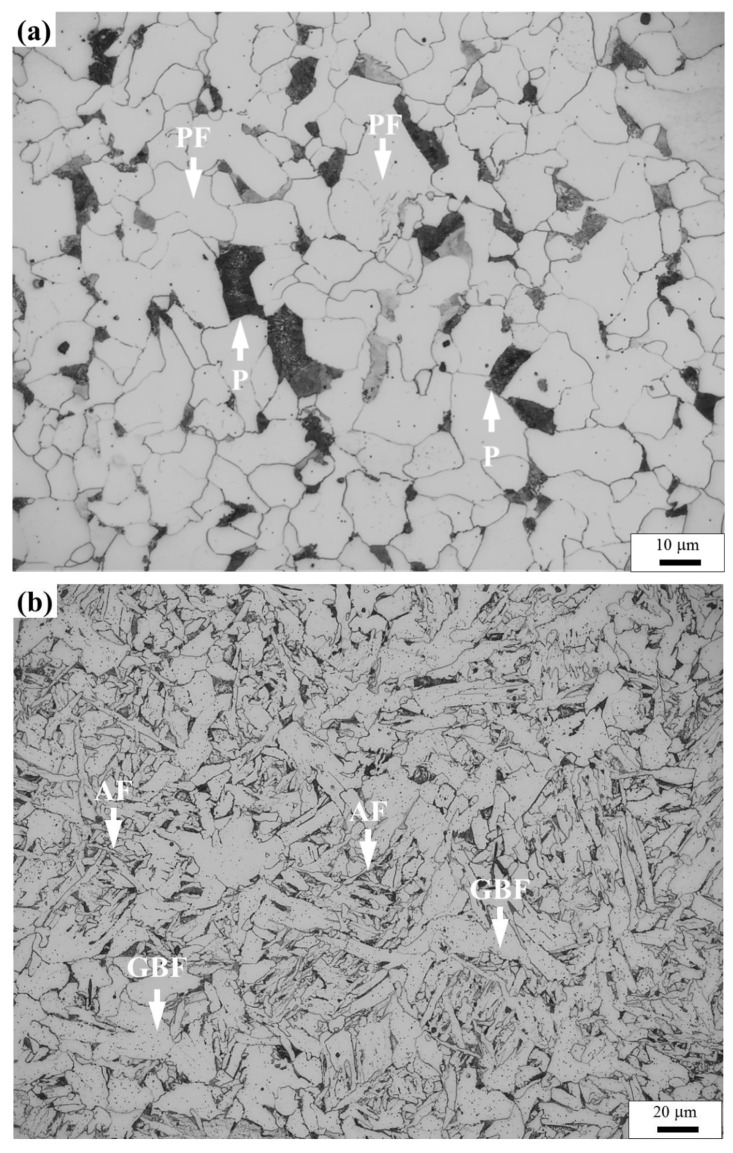

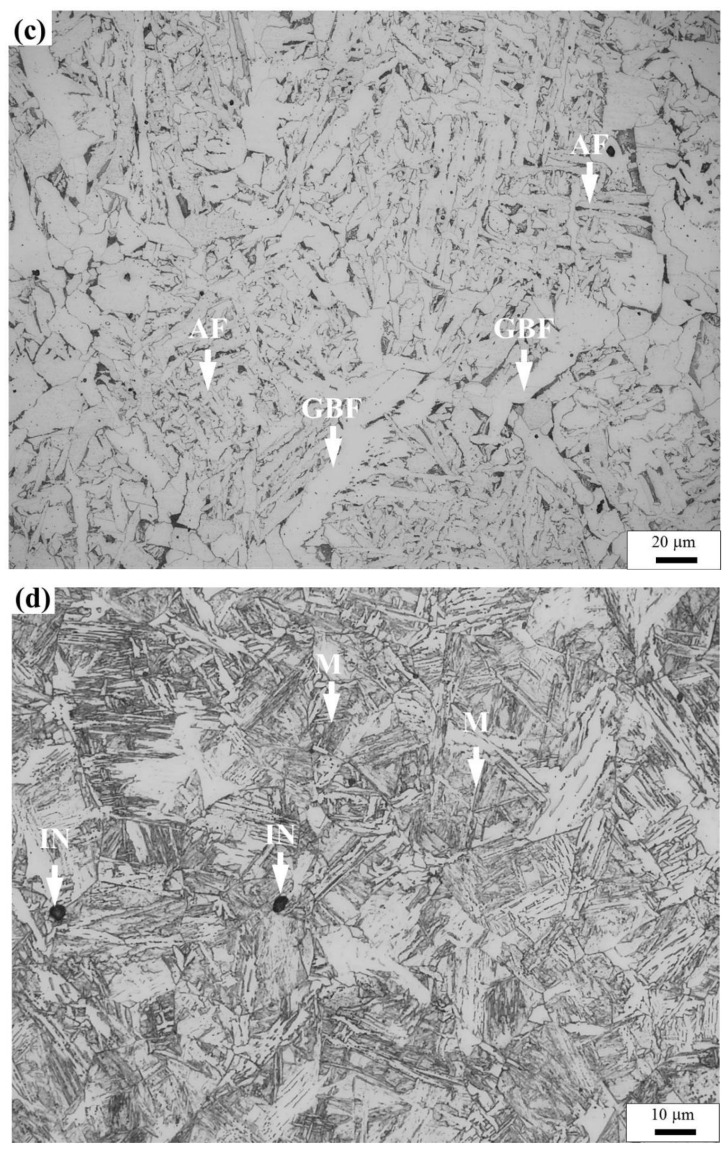

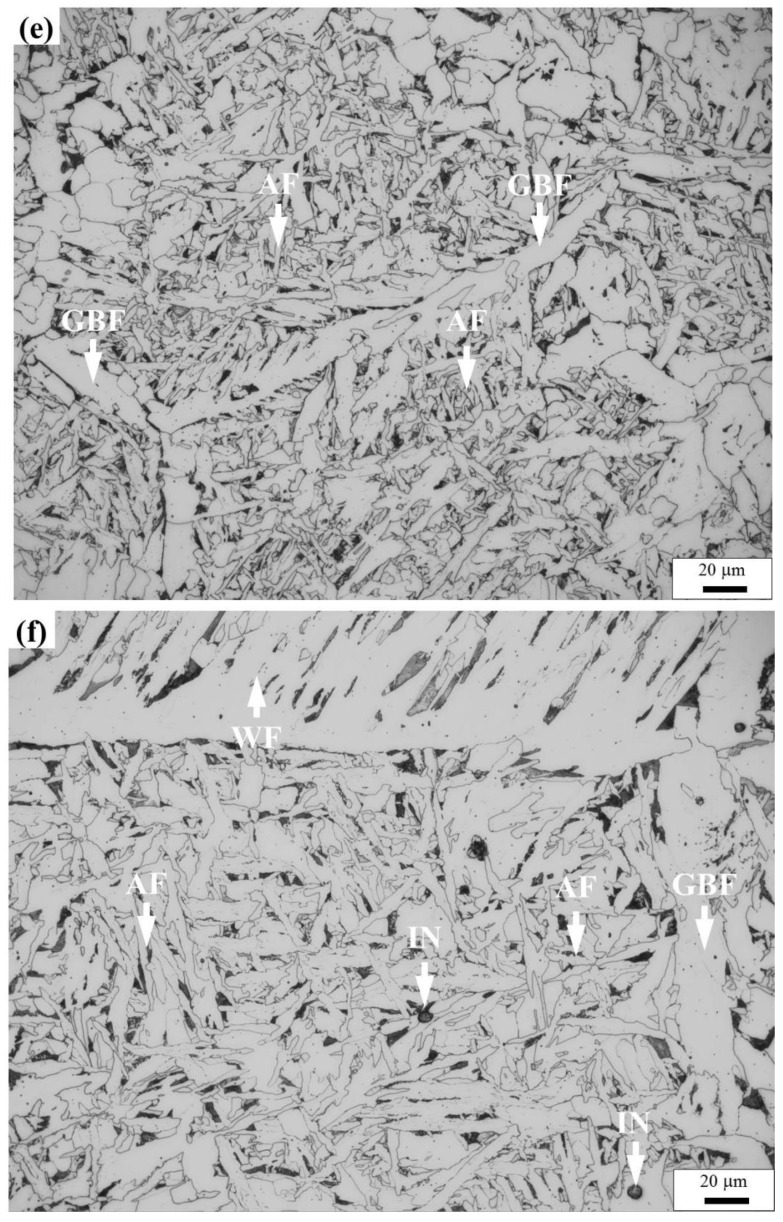
Typical microstructure of air-cooled base metal (**a**), CGHAZ with 100 kJ/cm in air-cooled initial microstructure (**b**), and CGHAZ with 400 kJ/cm in air-cooled initial microstructure (**c**), water-quenched base metal (**d**), CGHAZ with 100 kJ/cm in water-quenched initial microstructure (**e**), and the CGHAZ with 400 kJ/cm in water-quenched initial microstructure (**f**). P indicates pearlite, which is the back-etching grains; PF indicates polygonal ferrite, which mostly are equiaxed grains; M indicates martensite, which are thin laths; AF indicates acicular ferrite, which is ferrite that formed by combination of shear and displacive mechanism as sharp needles; GBF indicates grain boundary ferrite; WF indicates Widmanstatten ferrite; and IN indicates inclusions.

Figure 2 shows the microstructure for the air-cooled base metal and water-quenched base metal and the CGHAZ with heat input of 100 kJ/cm and 400 kJ/cm. The microstructure for the air-cooled base metal is composed of polygonal ferrite and pearlite, as shown in Figure 2a. The main microstructure constituent for the water-quenched base metal is martensite, as shown in Figure 2d. The microstructure for the CGHAZ with heat input of 100 kJ/cm mainly consists of grain boundary ferrite and acicular ferrite in both the air-cooled and water-quenched initial microstructure. The average size of grain boundary ferrite in the CGHAZ of the water-quenched initial microstructure is larger than that of the CGHAZ of the air-cooled initial microstructure. As the heat input is increased to 400 kJ/cm, the microstructure has coarsened in CGHAZ. The formation of acicular ferrite on inclusions and Widmanstatten ferrite nucleated around grain boundaries is observed in the CGHAZ of the water-quenched initial microstructure, as shown in Figure 2f.

**Figure 3:**
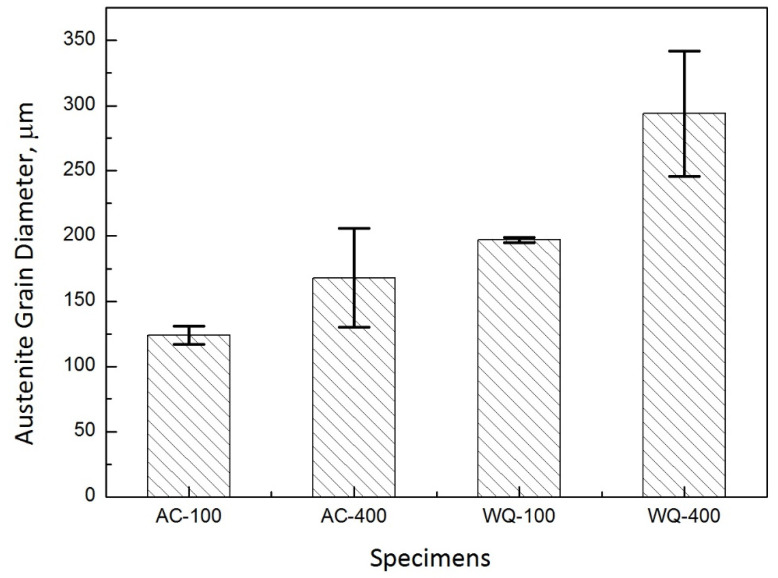
Prior austenite grain diameters in the CGHAZ with heat input of 100 kJ/cm and 400 kJ/cm. AC-100 is CGHAZ with 100 kJ/cm in air-cooled initial microstructure; AC-400 is CGHAZ with 400 kJ/cm in air-cooled initial microstructure; WQ-100 is CGHAZ with 100 kJ/cm in water-quenched initial microstructure; WQ-400 is CGHAZ with 400 kJ/cm in water-quenched initial microstructure.

Figure 3 shows the prior austenite grain diameters of the CGHAZ measured from the low magnification optical micrographs after welding thermal cycles. The austenite grain diameter in the CGHAZ of 100 kJ/cm and 400 kJ/cm in air-cooled initial microstructure is 124 ± 7, 168 ± 38 micrometers, respectively. For water-quenched initial microstructure, the austenite grain diameter for the CGHAZ of 100 kJ/cm and 400 kJ/cm is 197 ± 2, 299 ± 48 micrometers, respectively. Prior austenite grain diameter increased as the heat input is increased.

**Figure 4:**
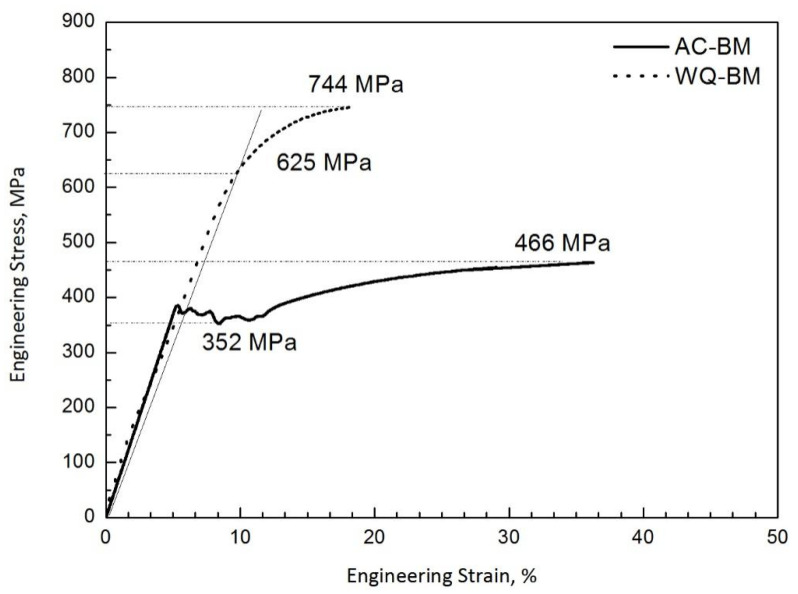
Stress–strain curves for the specimens with air-cooled base metal (AC-BM) and water-quenched base metal (WQ-BM).

Stress–strain curves for the air-cooled base metal and water-quenched base metal in TMCP-ed condition are shown in Figure 4. The yield to tensile strength ratio is identified for the air-cooled base metal and water-quenched base metal. For the air-cooled base metal, the stress–strain curve shows the upper and lower yield points. Its yield strength, ultimate tensile strength, elongation, and yield to tensile strength ratio are 352 MPa, 466 MPa, 28.5% and 0.76, respectively. For the water-quenched base metal, there is no yield plateau in the tensile curve; the yield strength is determined by the 0.2% strain offset method. Its yield strength, ultimate tensile strength, elongation, and yield to tensile strength ratio are 625 MPa, 744 MPa, 7.0% and 0.84, respectively. It is obvious that the water-quenched base metal is stronger, but the ductility is much lower than that of the air-cooled base metal.

**Figure 5:**
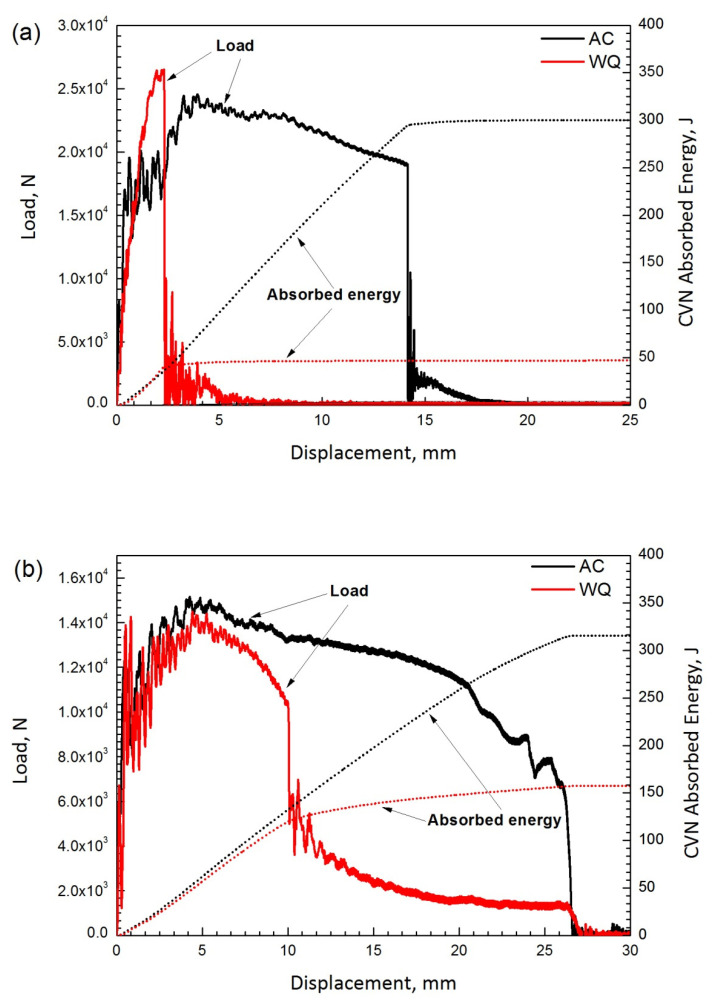

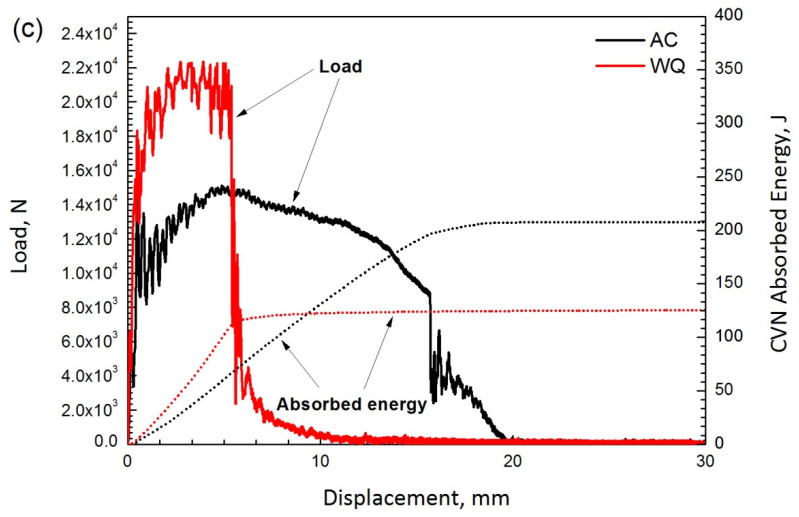
Typical load and absorbed energy versus displacement plots of base metal and CGHAZ by instrumented impact test at −20 °C. (**a**) base metal, (**b**) CGHAZ with 100 kJ/cm, (**c**) CGHAZ with 400 kJ/cm. AC indicates the air-cooled initial microstructure; WQ indicates the water-quenched initial microstructure.

Figure 5 shows typical plots of load and absorbed energy versus displacement from the instrumented Charpy impact test for base metal and the CGHAZ. The total absorbed energy (E_t_), consisting of the crack initiation energy (E_i_) and crack propagation energy (E_p_), is the area below the load–displacement curve. The crack initiation energy and crack propagation energy were determined by the area below the curve before and after the peak load, respectively [22]. Table 2 shows the absorbed energy data for the base metal and CGHAZ measured at −20 °C. The total energy E_t_ for the air-cooled base metal (AC-BM) is six times greater than that of the water-quenched base metal (WQ-BM). The total energy E_t_ for the CGHAZ of air-cooled initial microstructure is always higher than that of the CGHAZ of water-quenched initial microstructure. The total energy for the CGHAZ of the air-cooled initial microstructure is almost identical to its base metal; the total energy for the CGHAZ in the water-quenched initial microstructure, however, significantly increases from its base metal. For the effect of heat input, the total energy for the CGHAZ of either types of initial microstructure has decreased with an increasing heat input.

**Figure 6:**
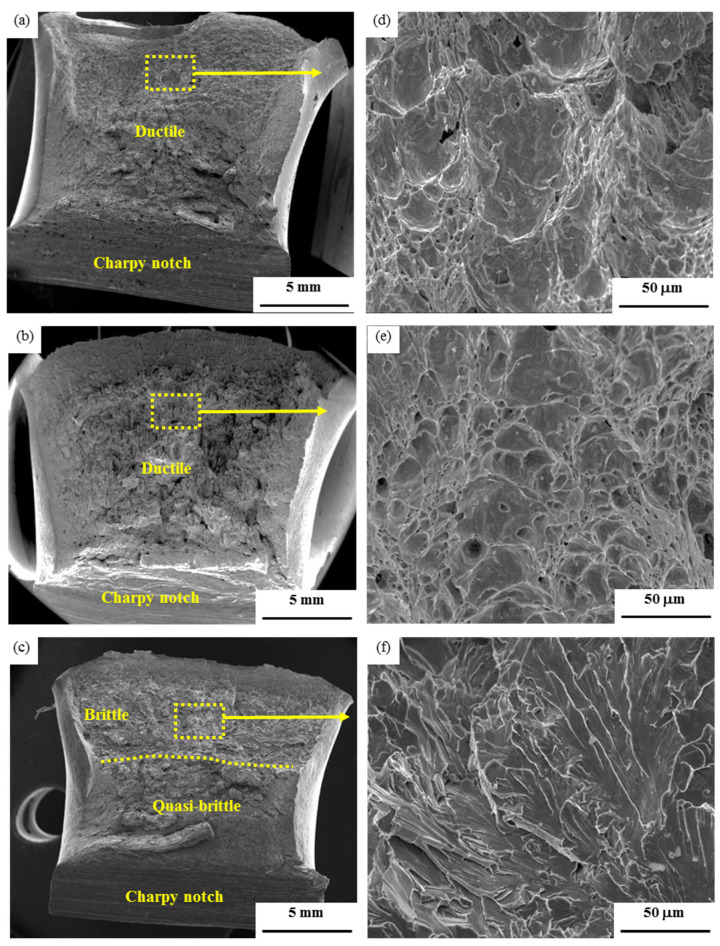
SEM fractographs of Charpy impact tested at −20 °C of the base metal (**a**,**d**), CGHAZ with 100 kJ/cm (**b**,**e**), CGHAZ with 400 kJ/cm (**c**,**f**) of the air-cooled initial microstructure.

**Figure 7:**
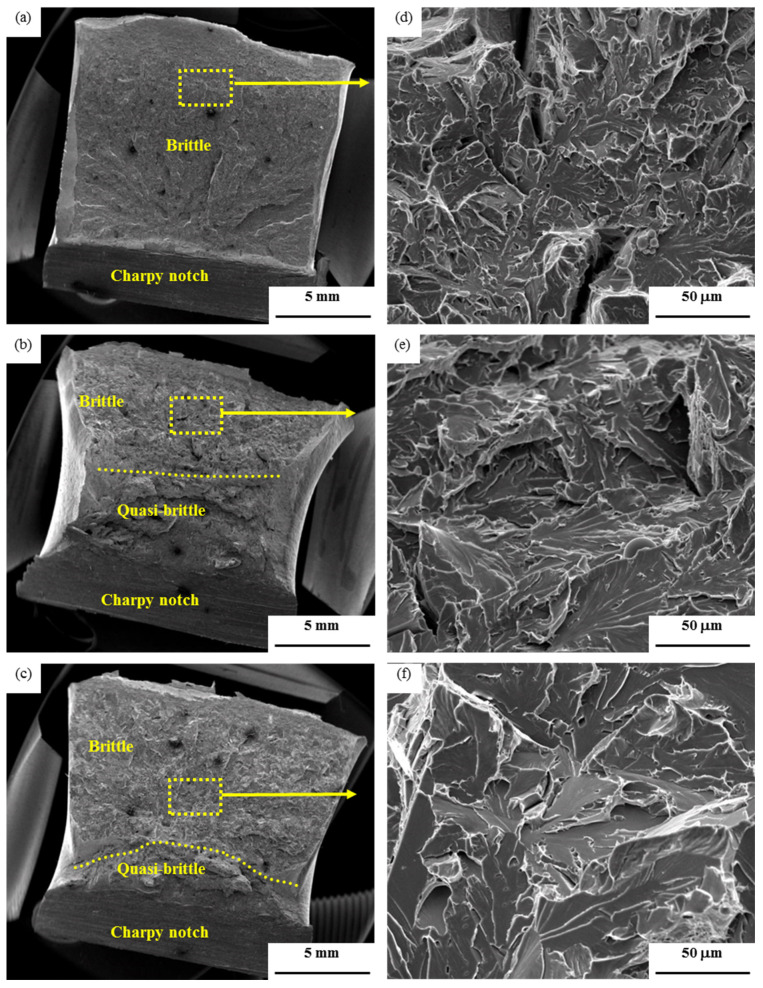
SEM fractographs of specimens Charpy impact tested at −20 °C of the base metal (**a**,**d**), CGHAZ with 100 kJ/cm (**b**,**e**), CGHAZ with 400 kJ/cm (**c**,**f**) of the water-quenched initial microstructure.

Figure 6 and Figure 7 show the fractographs of Charpy impact tests of both base metals and their CGHAZ. For the air-cooled initial microstructure, the base metal and the CGHAZ with the heat input of 100 kJ/cm show the ductile feature of fracture characterized by the dimples, with the size of dimples in the CGHAZ being appreciably smaller than the base metal, as shown in Figure 6a,b,d,e. A mixed micromechanism of fracture is presented in the CGHAZ of 400 kJ/cm heat input, which is characterized by a mixture of cleavage and micro-void coalescence, as shown Figure 6c,f. For the water-quenched initial microstructure, the base metal shows brittle fracture characterized by cleavage. However, the CGHAZ with heat input of 100 kJ/cm and 400 kJ/cm indicate a mixed micromechanism of fracture. The area of brittle zone for the CGHAZ of the water-quenched initial microstructure is larger than that of the CGHAZ of the air-cooled initial microstructure.

It is noted that for all Charpy tested samples, the fracture micromechanism at the root of notches, where the fracture initiates, is quasi-brittle, even for the water-quenched base metal (Figure 7a). This explains the 40 J crack initiation energy E_i_ for the water-quenched base metal. The crack propagation energy E_p_, however, depends on the fracture micromechanism at centers of Charpy cross-sections. Cleavage fracture gives E_p_ of 4 J for the water-quenched base metal. Mixed micromechanism fracture gives E_p_ of 65 to 96 J for the CGHAZ of the water-quenched initial microstructure. Ductile fracture gives E_p_ of 186 to 207 J for the air-cooled base metal and CGHAZ with heat input of 100 kJ/cm. Mixed micromechanism of fracture gives E_p_ of 147 J for the CGHAZ with heat input of 400 kJ/cm in the air-cooled initial microstructure. The hardness for the air-cooled base metal is lower than that of the water-quenched base metal. The hardness decreases with an increase in the heat input. For the air-cooled initial microstructure, a lower heat input (100 kJ/cm) has an impact toughness in the CGHAZ equal to that of the AC-MB base metal. In contrast, for water-quenched initial microstructure, a lower heat input (100 kJ/cm) has produced a significantly improved impact toughness in the CGHAZ compared with the WQ-BM base metal. For both initial base metal microstructures, an increase in heat input to 400 kJ/cm has the effect of lowering the impact toughness of the CGHAZ.

## 4. Discussion

**Figure 8:**
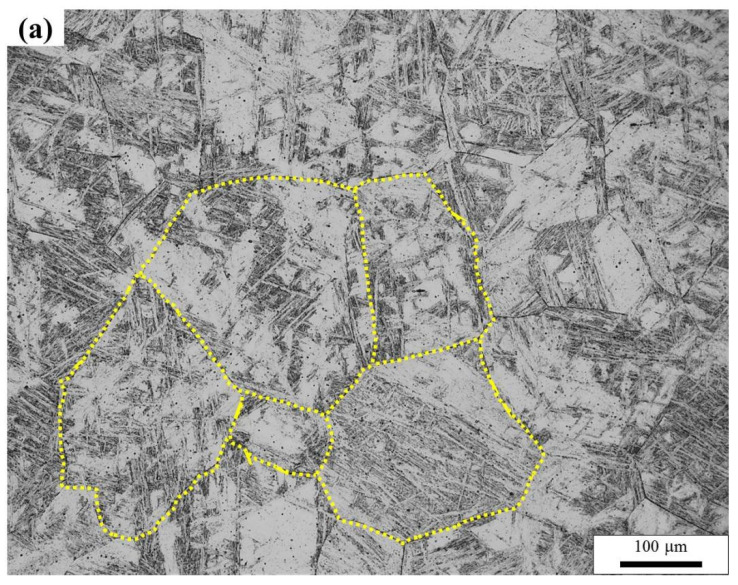

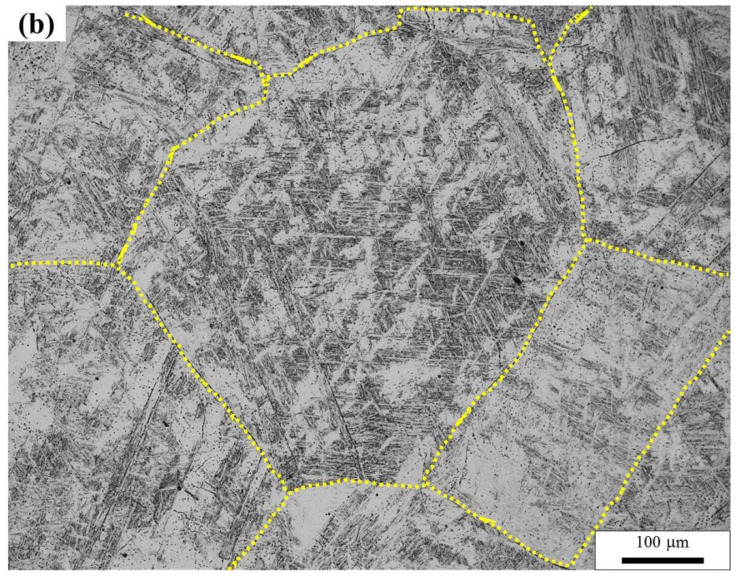
Prior austenite grains following a peak temperature of 1300 °C quenching by water for the air-cooled initial microstructure (**a**), for the water-quenched initial microstructure (**b**).

Based on the results presented above, it is clear that all the tested specimens share the identical chemical composition, process history, and microstructure in the coarse-grained heat affected zone except the initial starting microstructure. After welding using the same heat input, significant differences are obtained in the Charpy impact energy at −20 °C. The CGHAZ of air-cooled initial microstructure is significantly tougher than that of the water-quenched initial microstructure (Table 2). The possible reason for the difference in toughness behavior could be due to the difference in prior austenite grain size when different starting microstructures were heated to the peak temperature of CGHAZ. To understand the kinetics of austenite formation, both the air-cooled and water quenched base metals were heated to 1300 °C with a heating rate of 1 °C/s, held for 1s, followed by quenching with water and were then cooled by gas at cooling rate of 2.2 °C/s (it is close to the cooling rate from 800–500 °C with a heat input of 100 kJ/cm) using dilatometry. Figure 8 shows the microstructure of the air-cooled and water-quenched base metals where it is evident that the average austenite grain size for the air-cooled initial microstructure (197 ± 28 μm) was appreciably smaller than that of the water-quenched initial microstructure (273 ± 43 μm).

**Figure 9:**
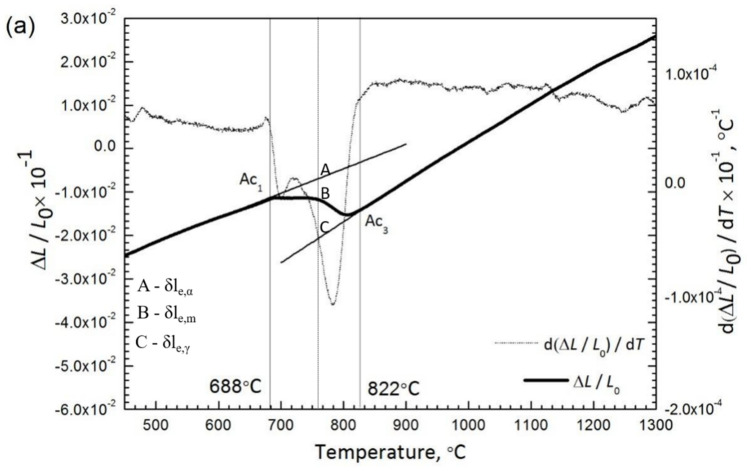

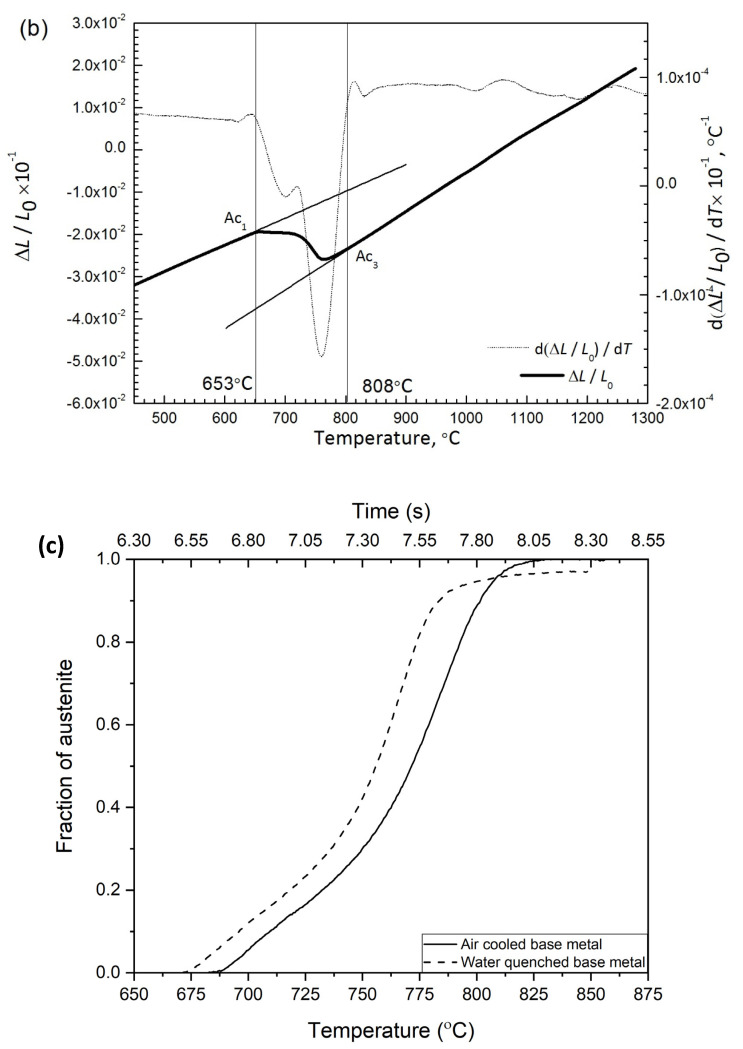

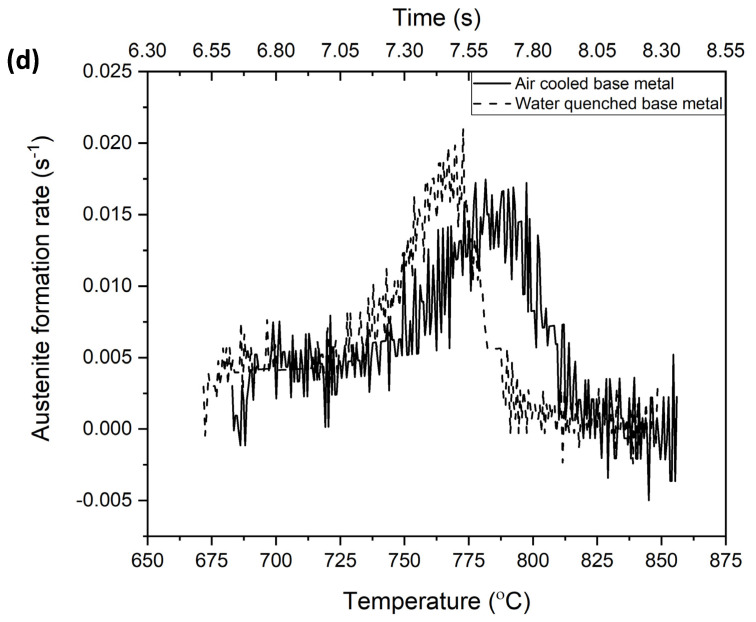
On heating dilatation and the corresponding derivative for the air-cooled initial microstructure (**a**), and for the water-quenched initial microstructure (**b**), and (**c**) austenite phase fraction and transformation (**d**) for air-cooled and water-quenched initial microstructure.

The dilation of each specimen ΔL was obtained as a function of temperature and time on continuous heating as shown in Figure 9. Based on the data, the dilatometry curve ΔL/L_0_ and the first derivative with respect to temperature d (ΔL/L_0_)/dT were plotted for both specimens during heating. The starting temperature of austenite transformation (A_c1_) for the air-cooled initial microstructure (688 °C) (Figure 9a) is higher than that of the water-quenched initial microstructure (653 °C) (Figure 9b). Corresponding phase fraction evolution and the rate of phase transformation of ferrite to austenite, obtained by taking numerical derivative of fraction of austenite formed at each time step (since the fraction of austenite is a dimensionless number, the formation rate of austenite is expressed as (s^−1^)), are shown in Figure 9c and Figure 9d, respectively. The phase fraction of austenite was estimated from dilatometry using lever rule by linear extrapolation of ferrite phase field dilation and austenite phase field dilation beyond the A_c1_ and A_c3_ temperature, respectively, and applying lever rule in the A_c1_ A_c3_ range to calculate the austenite phase fraction using the equation below:

$$f_{\gamma ,T}=\frac{{\delta l}_{e,\alpha }-{\delta l}_{m}}{{\delta l}_{e,\alpha }-{\delta l}_{e,\gamma }}$$

where δl_e, α_, δl_e,γ,_ δl_m_ are the extrapolated dilation from the ferrite phase field, extrapolated dilation from the austenite phase field, and the experimental dilation for given temperature.

On heating, austenite transformation happens in the air-cooled initial microstructure at a higher Ac_1_ temperature, leading to smaller prior austenite grain size compared with water-quenched initial microstructure for the same peak heating temperature. It should also be noted that the rate of formation of austenite from a martensitic starting microstructure is faster than the rate of formation of austenite from a polygonal ferritic starting microstructure.

**Figure 10:**
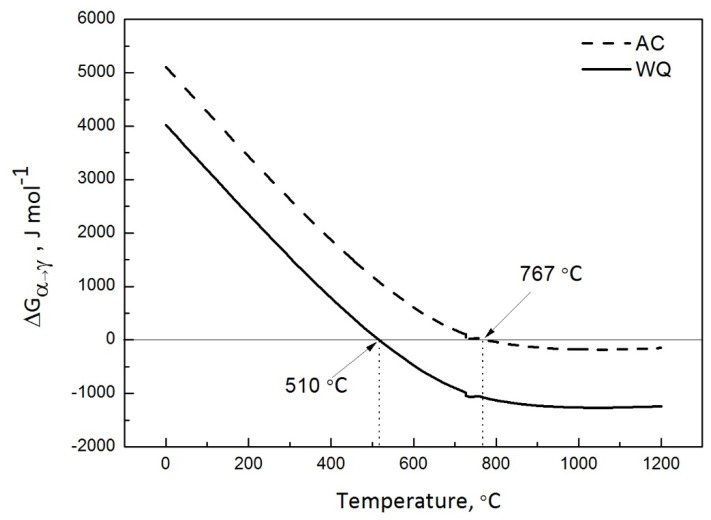
Driving force (molar Gibbs free energy change) for on-heating ferrite to austenite transformation calculated using ThermoCalc. AC is for air-cooled initial microstructure; WQ is for water-quenched initial microstructure.

To understand the reason for such an effect of initial microstructure on Ac_1_ and Ac_3_, the driving force for austenite transformation on heating was calculated using ThermoCalc. Since the water-quenched initial microstructure was subjected from water quenching after hot rolling, the initial microstructure is martensite with associated strain energy component, the strain energy values were added to ferrite (to better represent martensite) when performing the calculations [23,24]. It can be seen that under the equilibrium condition, the drive force (positive Gibbs free energy change) for austenite transformation in air-cooled initial microstructure is lower than that of the water-quenched initial microstructure, shown in Figure 10. The driving force calculations seem to indicate that the initial microstructure with ferrite plus pearlite microstructure is more stable compared with the martensitic microstructure. This result can be supported by the austenite formation activation energy reported by Edgar et al. [25]. They have studied the effect of initial microstructure on austenite formation kinetics in high-strength experimental microalloyed steels. They reported that the activation energy for the initial microstructure comprising bainite–martensite (530 kJ/mol) is lower than that of the ferrite–martensite/austenite initial microstructure (545 kJ/mol).

**Figure 11:**
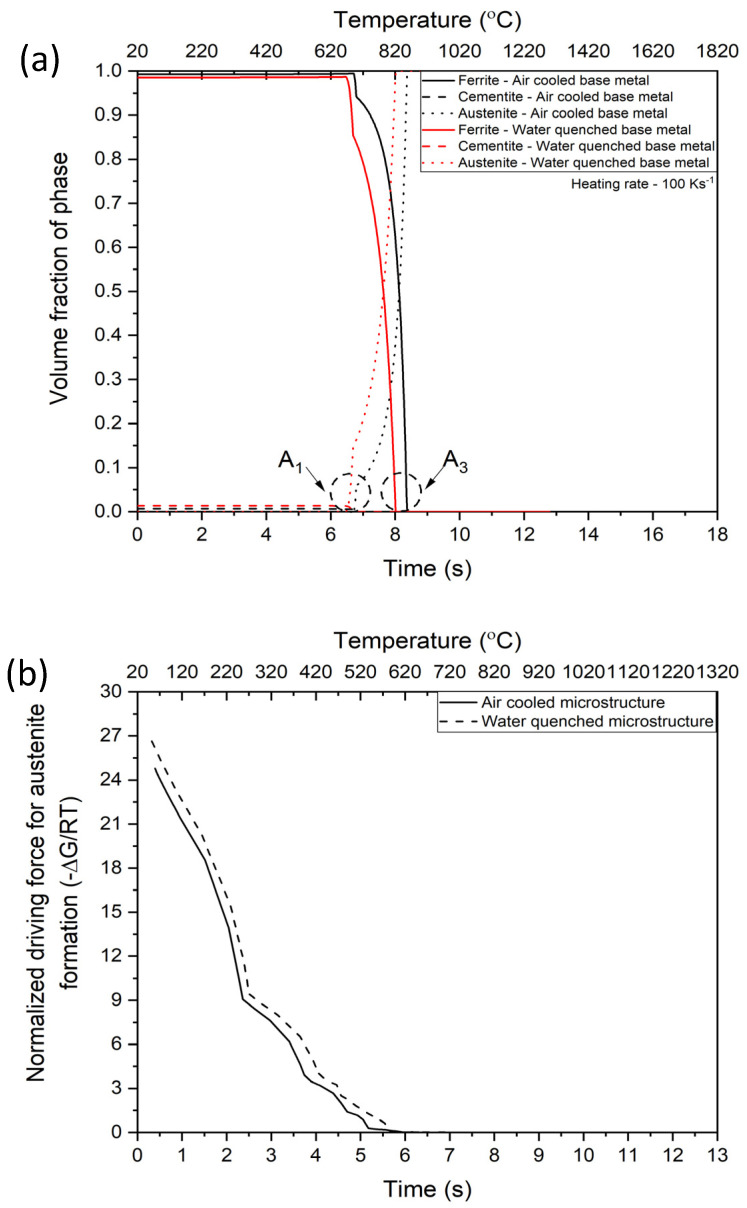

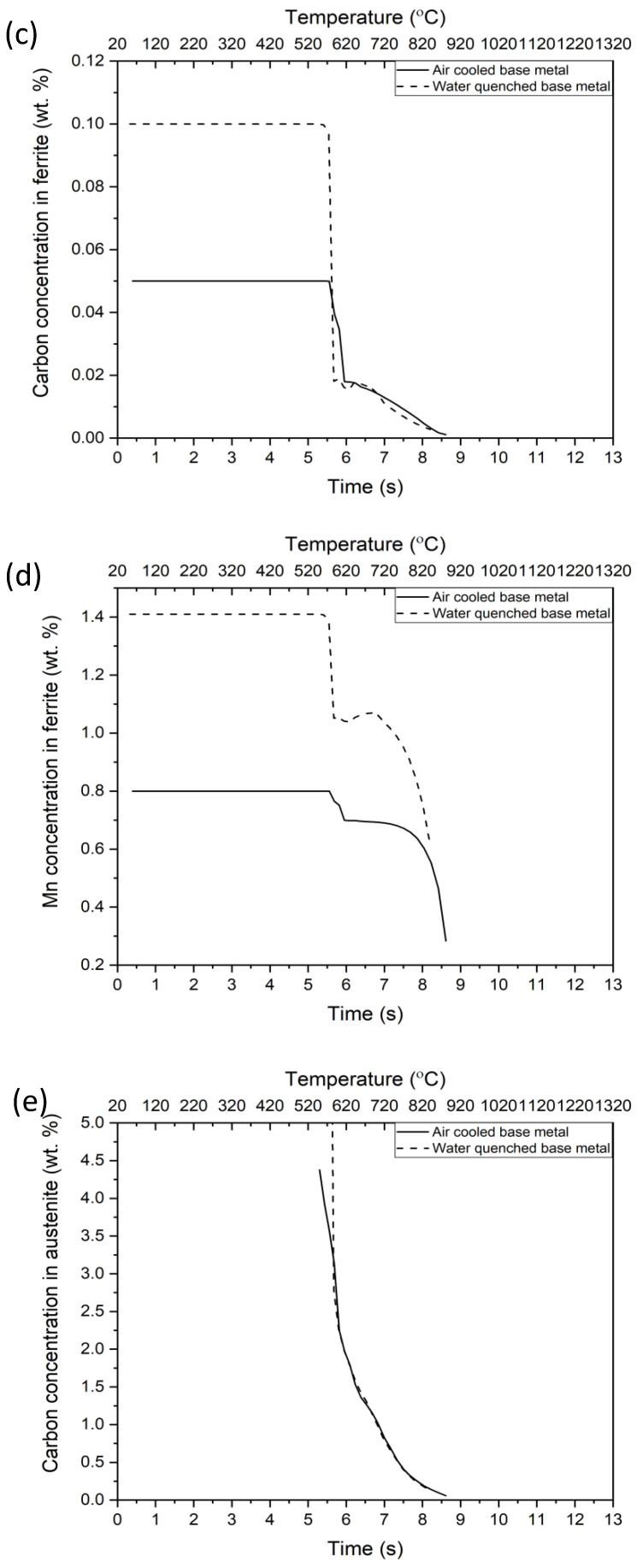

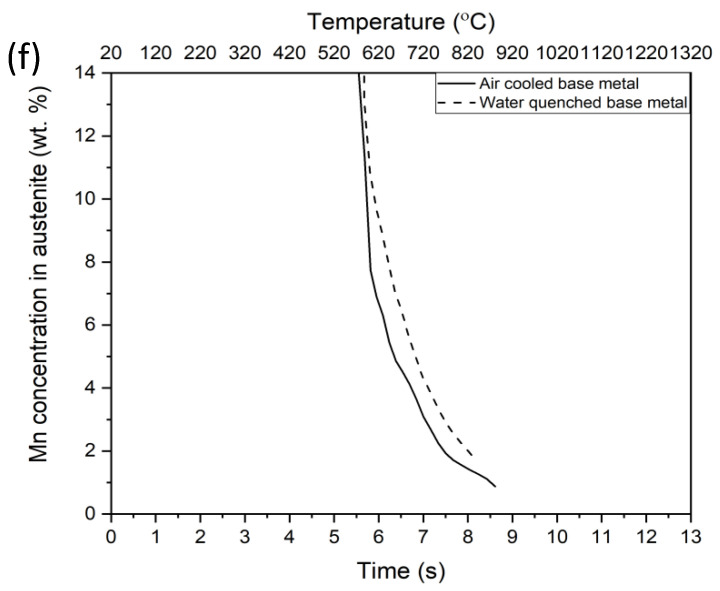
DICTRA and PRISMA calculations to understand the kinetics of austenite formation during CGHAZ simulation with different starting microstructures. (**a**) Evolution of phases as a function of time for the air-cooled and water-quenched starting microstructure, (**b**) normalized driving force for austenite formation as a function of temperature, (**c**) C concentration in ferrite as a function of transformation time, (**d**) Mn concentration in ferrite as a function of transformation time, (**e**) C concentration in austenite as a function of transformation time, and (**f**) Mn concentration in austenite as a function of transformation time.

To qualitatively understand the kinetics of austenite formation, DICTRA and PRISMA calculations were carried out as shown in Figure 11. For the formation of austenite from air-cooled based metal microstructure, the setup of the model consisted of grain size estimated from the micrographs, and composition estimated from equilibrium calculations from ThermoCalc. Austenite was allowed to form under local equilibrium conditions at the ferrite grain boundaries under experimental heating rate (100 °C/s). Since the fraction of pearlite is negligible in the air-cooled microstructure, nucleation of austenite from ferrite is considered in the present case. For the formation of austenite from water quenched base metal, the setup of the model consisted of grain size/martensitic lath size estimated from the micrographs. The composition for the water quenched based metal was taken to be the nominal composition of the alloy with the assumption that prior austenite transforms into martensite in a displacive manner without redistribution of solutes. Austenite was allowed at martensitic lath boundaries under local equilibrium conditions at experimental heating rate of (100 °C/s).

**Figure 12:**
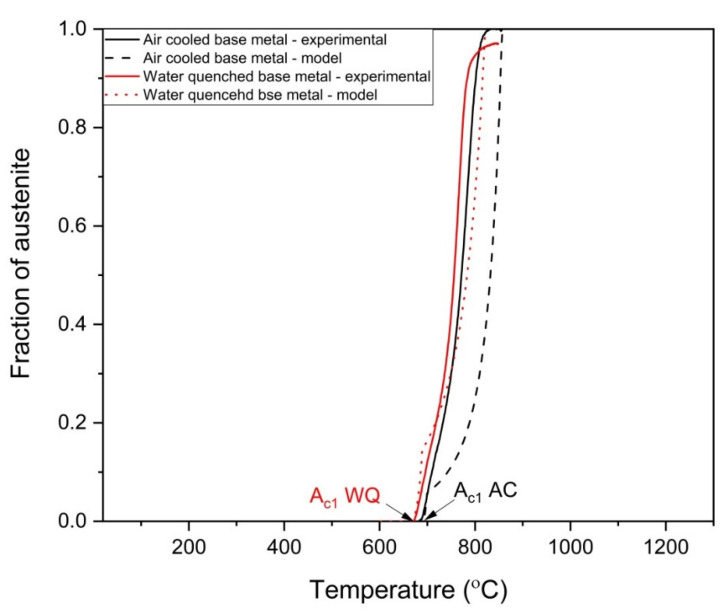
Comparison between experimental austenite formation and model predicted austenite formation.

Figure 11a shows the phase fraction evolution as a function of transformation time for air-cooled and water-quenched base metals predicted from DICTRA. It can be seen that the A_1_ and A_3_ temperature for water-quenched base metals is lower than the air-cooled base metal corroborating the experimental measurements from dilatometry in Figure 9. It should also be noted that the rate of formation of austenite is faster in the water-quenched base metal (slope of austenite formation line in Figure 11a) further corroborating with the experimental phase fraction rates in Figure 9. Figure 11b shows the normalized driving force for austenite formation in water-quenched and air-cooled base metals as a function of transformation time where it can be seen that the water-quenched base metal has a higher driving force for austenite formation compared with the air-cooled base metal which explains the higher rate of transformation to austenite. To understand the elemental partitioning behavior during the transformation, C and Mn profiles were extracted from the kinetic simulations. Figure 11c–f shows the concentration of C and Mn in air-cooled and water-quenched base metals in ferrite and austenite respectively. It can be seen that the diffusion of C and Mn in ferrite are the rate determining mechanisms for austenite formation. It can also be seen that the extent of enrichment of C and Mn (austenite stabilizers) at the interface is higher for the water-quenched base metal resulting in stabilizing austenite to a much greater extent in the water-quenched base metal than the air-cooled base metal, which is consistent with the results of Bhattacharyya et al. [26]. They showed that an increase in grain boundary mobility due to C enrichment increases the austenite grain boundary mobility by an order of magnitude compared with a decrease in grain boundary mobility due to Mn enrichment. As a result, the overall grain growth kinetics for water-quenched base metal are higher. It should be noted that the C and Mn profiles extracted from the kinetic simulations represent an estimate of C and Mn concentrations at the transformation interface. Experimental verification of the predicted concentration profiles are underway and the results will be published separately. To verify the phase fraction evolution predictions from the model and the experimental phase fraction evolution, austenite fraction evolution as a function of temperature is plotted in Figure 12.

It can be seen from Figure 12 that for both the water-quenched and air-cooled base metal, austenite nucleation is predicted by the model, however, there is some deviation in the experimental austenite fraction and austenite fraction predicted by the model during growth. One of the possible reasons could be that the model did not consider the effect of pearlite on the nucleation of austenite. Though the fraction of pearlite is much less compared with ferrite, austenite nucleates at the ferrite/pearlite boundaries and initially grows into the pearlite which can affect the overall fraction of austenite formed. This results in underprediction of austenite fraction. Future works will be carried out to fine tune the model including the effect of pearlite on austenite formation. Irrespective of the current limitations, the model predicts the A_c1_ and A_c3_ temperatures within reasonable error limits.

**Figure 13:**
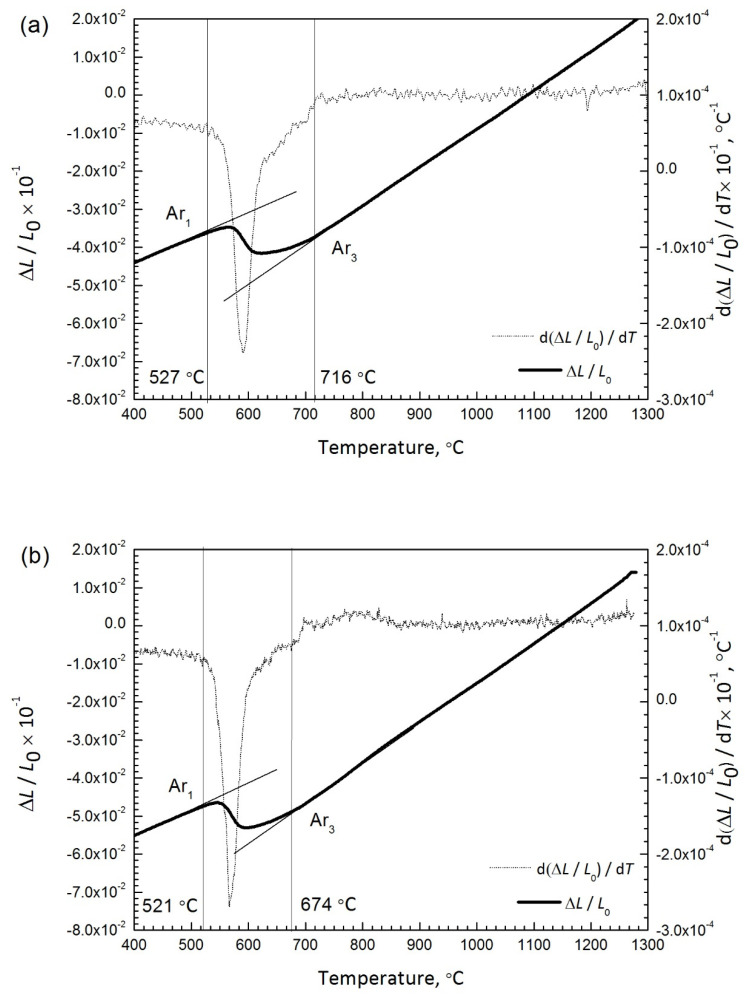
On cooling dilatation recordings and the corresponding derivative for the air-cooled initial microstructure (**a**), and for the water-quenched initial microstructure (**b**).

Apart from the austenite formation kinetics affecting the austenite grain size which in turn influences the toughness of the coarse-grained heat affected zone, the austenite formation also influences the on-cooling phase transformations, which can influence the toughness as well. On cooling, the phase transformation temperature (Ar_1_ and Ar_3_) is presented in Figure 13, at the cooling rate of 2.2 °C/s. The starting temperature of phase transformation (Ar_3_) for water-quenched initial microstructure (674 °C) is lower than that of the air-cooled initial microstructure (716 °C). The finishing temperature of phase transformation (Ar_1_) is almost the same for both specimens. It can be explained by the prior austenite grain size. The stability for the water-quenched initial microstructure is more than that of the air-cooled initial microstructure due to the water-quenched initial microstructure with larger prior grain size, leading to lower phase transformation temperature. Lan et al. [27] and Lee et al. [28] have investigated the effect of autenite grain size on the transformation temperature on cooling in microalloyed steels. They indicated that the fine austenite grain size can reduce the transformation temperature. Similar results can be obtained by an in situ observation result on the real welding-induced phase transformation reported by Elmer et al. [29]. They showed that the amount of untransformed austenite decreases gradually from the FGHAZ to the CGHAZ within a certain time at the cooling stage. It seems to be illustrated that more brittle microstructure, such as martensite, is available for the water-quenched initial microstructure than that of the air-cooled initial microstructure if subjecting it to the same thermal cycle, resulting in the lower toughness.

## 5. Conclusions

In this study, the effect of initial base metal microstructure on the impact toughness of the CGHAZ during high heat input welding was studied, and the following conclusions can be made:

(1) The specimens for the water-quenched initial microstructure are stronger than that of the air-cooled initial microstructure. The average Charpy impact energy for the CGHAZ with heat input of 100 kJ/cm and 400 kJ/cm at testing temperature of −20 °C for the air-cooled initial microstructure is higher than that of the water-quenched initial microstructure.
(2) The prior austenite grain diameters of the CGHAZ for the air-cooled initial microstructure are smaller than that of the water-quenched initial microstructure. The A_c1_ temperature for the air-cooled initial microstructure is higher than that of the water-quenched initial microstructure. Thermodynamic and kinetic calculations in combination with dilatometry indicate that the air-cooled initial microstructure has a lower driving force for austenite formation, and therefore a higher A_c1_ temperature, which resulted in a smaller prior austenite grain diameter in the air-cooled initial microstructure compared with the water-quenched initial microstructure.
(3) The A_r3_ temperature for the air-cooled initial microstructure is higher than that of the water-quenched initial microstructure. The stability for the water-quenched initial microstructure is more than that of the air-cooled initial microstructure because the water-quenched initial microstructure has a larger prior grain size, which has led to a lower phase transformation temperature.

## Figures and Tables

**Table 1:** Chemical composition of the experimental steel (mass%).

C	Si	Mn	P	S	Ti	Zr	O	N
0.1	0.18	1.41	<0.008	<0.005	0.014	0.01	0.0016	0.0039

**Table 2:** Charpy impact energies and hardness for base metal and CGHAZ at −20 °C.

Specimen	E_t_, J	E_i_, J	E_p_, J	Hardness, HV
AC-BM	257 ± 37	71 ± 4	186 ± 34	169 ± 6
AC-100	259 ± 52	51 ± 3	207 ± 49	219 ± 9
AC-400	209 ± 7	62 ± 3	147 ± 4	205 ± 4
WQ-BM	42 ± 4	39 ± 2	4 ± 2	294 ± 20
WQ-100	148 ± 27	53 ± 3	96 ± 24	218 ± 14
WQ-400	118 ± 8	53 ± 2	65 ± 6	208 ± 10

E_t_ (total energy) = E_i_ (crack initiation energy) + E_p_ (crack propagation energy); AC-BM: air-cooled base metal, AC-100: CGHAZ with 100 kJ/cm in air-cooled initial microstructure, AC-400: CGHAZ with 400 kJ/cm in air-cooled initial microstructure, WQ-BM: water-quenched base metal, WQ-100: CGHAZ with 100 kJ/cm in water-quenched initial microstructure, and WQ-400: CGHAZ with 400 kJ/cm in water-quenched initial microstructure.

## Data Availability

Not applicable.
